# Saturated mutagenesis of ketoisovalerate decarboxylase V461 enabled specific synthesis of 1-pentanol via the ketoacid elongation cycle

**DOI:** 10.1038/s41598-017-11624-z

**Published:** 2017-09-12

**Authors:** Grey S. Chen, Siang Wun Siao, Claire R. Shen

**Affiliations:** Department of Chemical Engineering, National Tsing Hua University, Hsinchu, Taiwan

## Abstract

Iterative ketoacid elongation has been an essential tool in engineering artificial metabolism, in particular the synthetic alcohols. However, precise control of product specificity is still greatly challenged by the substrate promiscuity of the ketoacid decarboxylase, which unselectively hijacks ketoacid intermediates from the elongation cycle along with the target ketoacid. In this work, preferential tuning of the *Lactococcus lactis* ketoisovalerate decarboxylase (Kivd) specificity toward 1-pentanol synthesis was achieved via saturated mutagenesis of the key residue V461 followed by screening of the resulting alcohol spectrum. Substitution of V461 with the small and polar amino acid glycine or serine significantly improved the Kivd selectivity toward the 1-pentanol precursor 2-ketocaproate by lowering its catalytic efficiency for the upstream ketoacid 2-ketobutyrate and 2-ketovalerate. Conversely, replacing V461 with bulky or charged side chains displayed severely adverse effect. Increasing supply of the iterative addition unit acetyl-CoA by acetate feeding further drove 2-ketoacid flux into the elongation cycle and enhanced 1-pentanol productivity. The Kivd V461G variant enabled a 1-pentanol production specificity around 90% of the total alcohol content with or without oleyl alcohol extraction. This work adds insight to the selectivity of Kivd active site.

## Introduction

With genomic mining and protein engineering, biosynthesis of non-natural alcohols has made significant progress via expansion of iterative carbon chain elongation reactions naturally found in the reverse β-oxidation^1–3^ and the branched chain amino acid pathway^4–6^. Construction of artificial metabolism has allowed production of various aliphatic and aromatic alcohols synthetically upon enlargement of binding pocket and find-tuning of substrate specificity of key enzymes^4–7^. As a semi-natural metabolite, the straight chain 1-pentanol has been detected from yeast fermentation along with other isoamyl alcohols in trace amounts^8^. Beyond its potential as the next generation fuel alternative with higher energy density and lower hygroscopicity, 1-pentanol is also used industrially as coating solvent and chemical feedstock in flavor formation.

**Figure 1:**
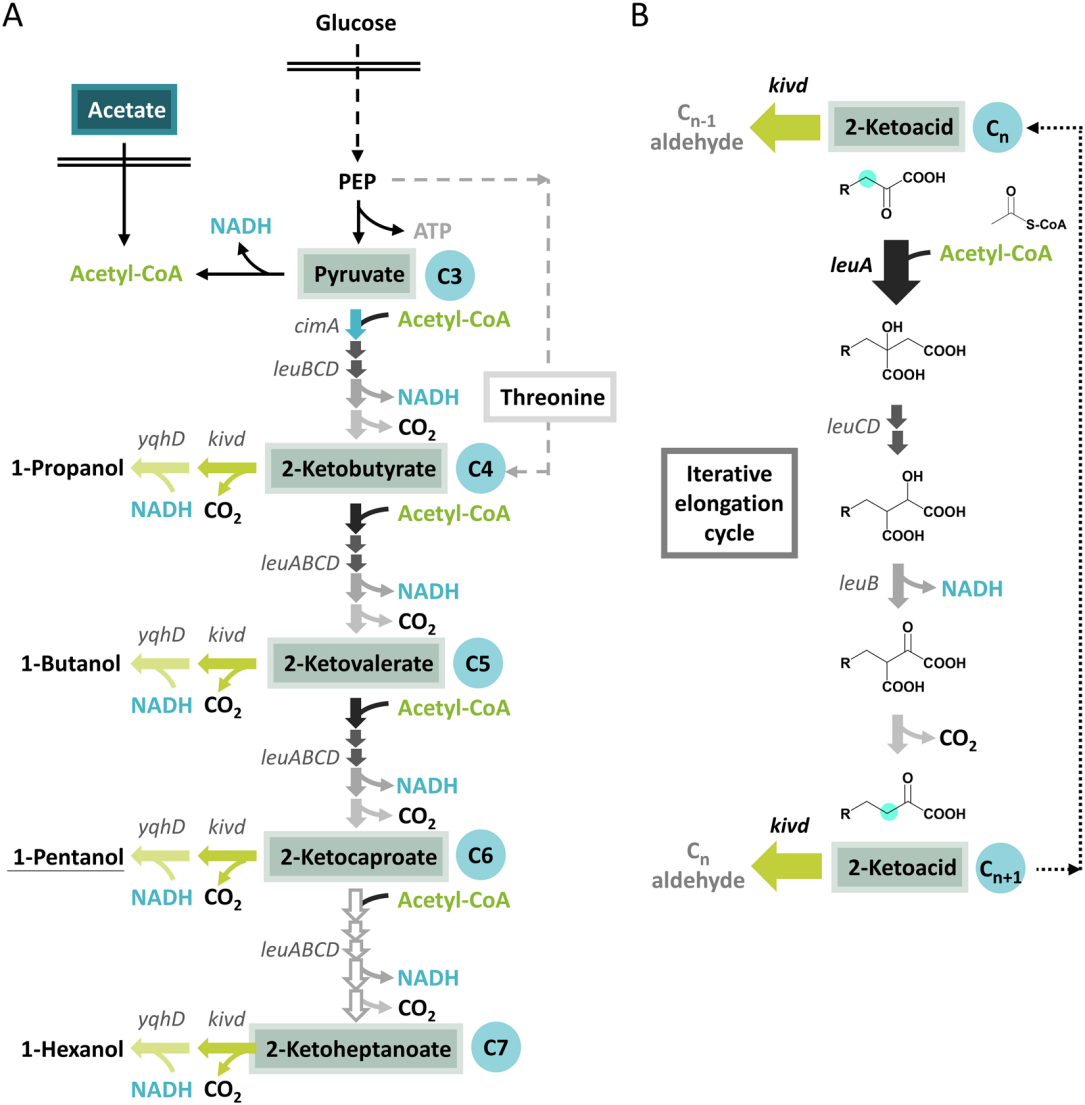
Schematics of alcohol production via the iterative ketoacid elongation. (**A**) Synthesis of straight chain alcohols from the different 2-ketoacids with various chain length. Number of carbons in each 2-ketoacid is highlighted by the blue circle. Formation of 2-ketobutyrate via the citramalate pathway (highlighted by blue block arrow) follows the identical reaction chemistry as the iterative ketoacid elongation except homologous enzyme CimA is used here instead of LeuA due to the limited substrate range of LeuA. Inefficiency of wild type LeuA to extend 2-ketocaproate further is illustrated by the hollow block arrows. Native activity of the threonine pathway for 2-ketobutyrate synthesis is shown by the gray dashed arrows. Enzymes Kivd and YqhD were overexpressed to decarboxylate and reduce the 2-ketoacid into corresponding alcohol. Feeding of acetate was performed to increase supply of the iterative addition unit acetyl-CoA. (**B**) Iterative elongation cycle catalyzed by enzymes LeuABCD. The competition between enzymes Kivd and LeuA decides the fate of 2-ketoacid intermediates: the 2-ketoacids can either escape the elongation cycle by decarboxylating into aldehyde (green block arrow) or re-enter the cycle (black block arrow). Acetyl-CoA serves as the repetitive addition unit where the 2-ketoacid is extended by one carbon at the β position (neon blue circle) after each cycle. PEP, phosphoenolpyruvate.

While utilization of the odd-chain reverse β-oxidation scheme resulted in a 1-pentanol titer around 0.1 g/L^2^, expansion of the ketoacid elongation pathway involved in leucine biosynthesis by rational design of key enzymes allowed production of 0.5–2 g/L of 1-pentanol coupled to secretion of many alcohol by-products (Fig. 1A) such as the intermediate 1-propanol and 1-butanol and the downstream 1-hexanol, accounting for a significant portion of the total alcohol content^4–6^. Although a wide variety of non-natural alcohols has been demonstrated by engineering the iterative ketoacid elongation, precise control of product specificity is still difficult to achieve due to substrate promiscuity of the enzymes catalyzing the terminal decarboxylation and reduction reaction. Thus, it is our goal in this work to increase the efficiency and specificity of 1-pentanol production in *Escherichia coli* by site-saturated mutagenesis of the terminal enzyme ketoacid decarboxylase.

Naturally found in leucine biosynthesis for the extension of 2-ketoisovalerate to 2-ketoisocaproate, the enzymes encoded by *leuABCD* catalyze the iterative elongation of 2-ketoacid using acetyl-CoA as the recursive addition unit, inserting one carbon at the β position after each round (Fig. 1B). Aside from its native substrate, the committing enzyme 2-isopropylmalate synthase (LeuA) was also observed to react efficiently with 2-ketobutyrate (2KB), leading to the unspecific synthesis of the non-natural amino acid norvaline^9^. It is interesting to note that the major gateway to repetitive ketoacid elongation appears to be controlled by LeuA while LeuBCD displayed great plasticity^4, 5^. Upon subsequent protein engineering, the broadened substrate acceptance of LeuA has allowed the generation of various synthetic 2-ketoacids and the corresponding higher chain alcohols up to C8 by coupling to the promiscuous ketoisovalerate decarboxylase Kivd from *Lactococcus lactis* and alcohol dehydrogenase ADH6 from *Saccharomyces cerevisiae* ^5, 6^. However, the substrate promiscuity of Kivd also led to inevitable production of the upstream alcohols by hijacking the 2-ketoacid precursors unspecifically before they re-enter the iterative elongation (Fig. 1A).

Depending on the relative affinity and catalytic activity of LeuA and Kivd toward a particular 2-ketoacid, the 2-ketoacid intermediate would either enter the fate of chain elongation or leave the elongation cycle and get converted into alcohol (Fig. 1). Thus, synthesis of higher chain alcohol such as 1-butanol via the ketoacid pathway has always been accompanied by secretion of 1-propanol derived from its direct precursor 2-ketobutyrate^10, 11^. Previously, formation of the upstream alcohol byproducts could be effectively lowered by spatial separation of Kivd and the 2-ketoacid intermediates via compartmentalization as demonstrated in *S*. *cerevisiae* ^12^. On the other hand, bioprospecting and mutagenesis of Kivd homologues^6^ also successfully skewed the straight-chain alcohol distribution from C3–C7 to C5–C8 by dropping the overall decarboxylation activity toward shorter 2-ketoacids. In particular, the V461A/F381L^4, 5^ and the G402V/M538L/F542V^6^ variants created by two independent studies demonstrated the most prominent enhancement in the Kivd specificity toward higher chain 2-ketoacids and achieved the synthesis of various branched and straight chain alcohols up to 1-octanol when coupled to LeuA mutants. While previous approaches integrating genomic mining, homology modeling, and computation-directed mutagenesis have shown great success toward expanding the artificial alcohol repertoire, here we aim to mine the potential of the key residue V461 for its ability to fine-tune Kivd selectivity toward 2-ketocaproate by saturated mutagenesis.

By screening Kivd V461 variants, a key residue previously shown to play significant role in the 2-ketoacid preference of Kivd, we successfully minimized the diversion of 2-ketoacid flux into intermediate alcohols 1-propanol and 1-butanol and improved the production specificity of 1-pentanol from 40% to 90% of the total alcohol content. Utilization of the wild type LeuA^FBR^ (feedback resistant) in the ketoacid elongation cycle also effectively suppressed the formation of downstream 1-hexanol due to its natural inefficiency to extend 2-ketocaproate further as demonstrated previously by *in vitro* study^5^. Supply of the essential precursor 2KB was achieved via overexpression of the citramalate pathway coupled to native activity of the threonine pathway synergistically. Here, saturated mutagenesis enabled us to examine the full potential of residue 461 in controlling the Kivd selectivity that may sometimes be overlooked or difficult to pinpoint by rational design. Although laborious, screening of saturated mutants provides complete information on the impact of a single residue toward a desired function. The positive Kivd variant V461G minimized diversion of flux into the upstream 1-propanol and 1-butanol and demonstrated superior specificity toward 1-pentanol synthesis, which was not achieved previously with the V461A substitution^4^. Kinetic characterization of the positive Kivd mutants revealed that the increase in 1-pentanol specificity was a result of decreased activity toward the upstream 2KB and 2-ketovalerate (2KV). Furthermore, external feeding of acetate to ensure adequate supply of the recursive addition unit acetyl-CoA for multiple rounds of ketoacid elongation was confirmed beneficial toward alcohol production for the first time in this work. With *in situ* extraction using oleyl alcohol, the final titer of 1-pentanol reached 4.3 g/L with complete abolishment of 1-propanol and 1-butanol level less than 4% of the total alcohol secreted. These strategies should be applicable to the engineering of artificial metabolism based on the iterative ketoacid elongation. This work not only demonstrates that Kivd specificity can be directly controlled by mutation of a single residue rather than double or tripled mutants as shown previously, but also helps building the foundation for further refining of Kivd selectivity based on the resulting alcohol spectrum of the 20 mutants.

## Results and Discussion

### Increasing LeuA expression to drive the ketoacid elongation cycle toward 2-ketocaproate

To develop a strain which synthesizes 1-pentanol with high efficiency and specificity, we must first ensure sufficient supply of 2KB and adequate expression of LeuA to channel carbon flux into two rounds of ketoacid elongation (Fig. 1A). As shown previously, biosynthesis of 2KB in *E*. *coli* can be achieved by overexpression of the native threonine pathway^11^ or the heterologous citramalate pathway upon evolution of the citramalate synthase (CimA) from *Methanococcus jannaschii* ^10^. Originated from their complementary nature, synergy of the threonine pathway and the citramalate pathway for 2KB synthesis was further demonstrated using 1-propanol production as a readout^13^, leading to higher yield and productivity of 1-propanol compared to the level obtained by each pathway individually. Therefore, overexpression of the citramalate pathway coupled to native activity of the threonine pathway was adopted in this case to ensure efficient supply of 2KB to initiate the elongation cycle. Once entered the elongation cycle, the relative activity of LeuA and Kivd toward each 2-ketoacid will determine the resulting alcohol spectrum. Examination of their *in vitro* catalytic efficiencies revealed that whereas wild type (WT) Kivd exhibits broad activity toward C4–C8 straight chain 2-ketoacids^6^, WT LeuA^FBR^ (feedback resistant variant G462D) displayed significant drop in activity starting at the C6 2-ketocaproate (2KC)^5^. The intrinsic limitation of WT leuA^FBR^ to extend 2KC further therefore is taken advantage of to terminate the ketoacid elongation cycle at the 1-pentanol stage. The NADPH-dependent aldehyde reductase encoded by *yqhD* from *E*. *coli* was used here toward alcohol production for its high solubility and broad substrate range demonstrated previously by various studies^14–18^.

**Figure 2:**
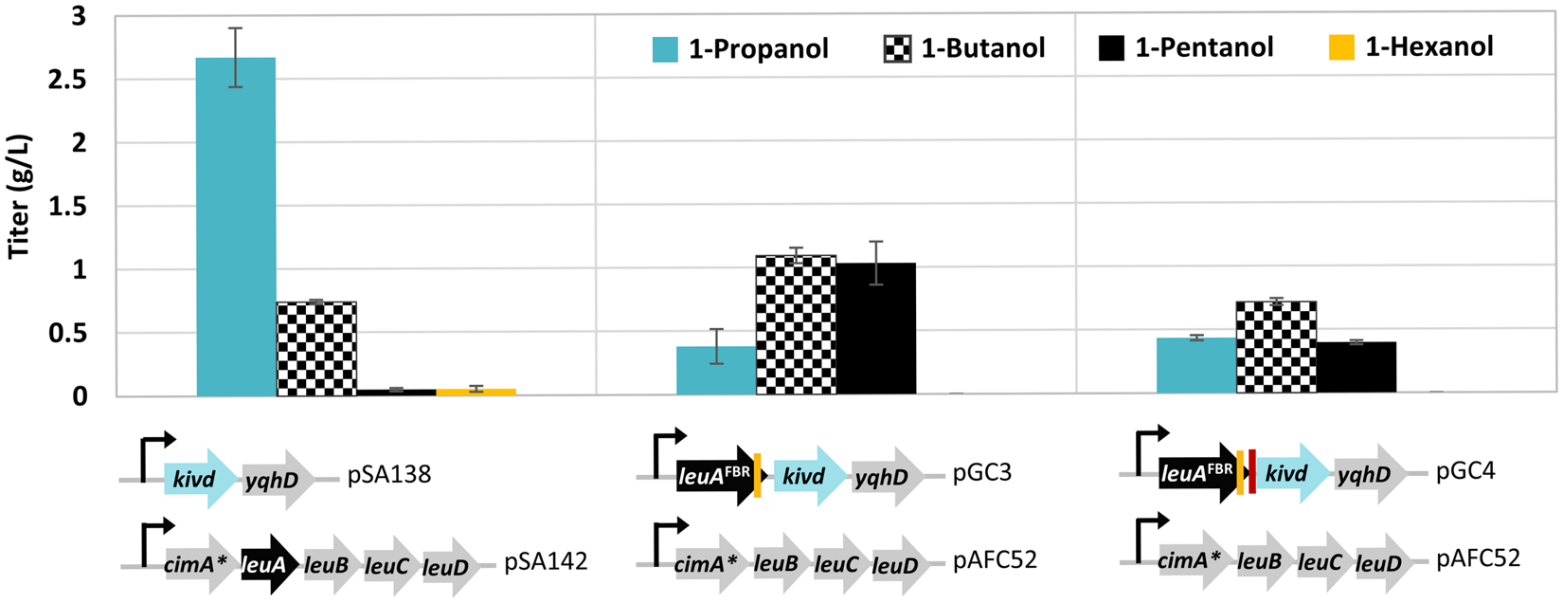
Increasing LeuA expression helped driving 2-ketobutyrate flux into 1-butanol and 1-pentanol. Strain CRS59 (Δ*ilvB* Δ*ilvI* Δ*leuA*) transformed with the different plasmid combination was used in this study. Modification of operon structure and the corresponding plasmid number are shown underneath the figure. The yellow band indicates the presence of native *leuB* RBS embedded at the end of *leuA*. The red band indicates the insertion of an additional synthetic RBS in front of *kivd*. Samples were taken after 48 h of induction. The error bars represent the standard deviation of three replicates.

Since the competition between LeuA and Kivd decides the fate of the intermediate 2-ketoacids (Fig. 1), we first set out to increase the expression of LeuA in the hope to enhance the rate of ketoacid elongation and lower the formation of upstream alcohol byproducts. Compared to the previous construct where LeuA was overexpressed from a medium copy plasmid behind CimA* (pSA142)^10^, here we cloned the feedback resistant LeuA^FBR^ (G462D) as the first gene downstream of P_L_lacO1 on a high copy plasmid (pGC3). Enzymes CimA* and the promiscuous LeuBCD remained overexpressed from the medium copy plasmid pAFC52. It is noted that the CimA* mutant Δ2^10^ was used in this study for its similar performance but less amino acid substitution compared to the mutant 3.7 harbored on plasmid pSA142. Since the primary focus of this study was to improve the specificity of straight chain alcohols synthesized via the elongation cycle by tuning Kivd selectivity for a particular chain length, the branched chain amino acid (BCAA) pathways were disrupted by the elimination of acetohydroxy acid synthase I and III encoded by *ilvB* and *ilvI*. Studies focusing on straight chain alcohols^5, 6^ have consistently taken this approach to completely abolish synthesis of methyl alcohols for better readout of Kivd activity toward straight chain 2-ketoacids. Strain CRS59 (Δ*ilvB* Δ*ilvI* Δ*leuA*) therefore was used here to eliminate competition of key enzymes and precursors by the BCAA pathway and to minimize background effect from the chromosomal *leuA*. During the design of synthetic operon on plasmid pGC3 (Table 1), we noticed the presence of a potential ribosomal binding site (RBS) embedded at the end of *leuA* coding region which originally drives the expression of *leuB* in *E*. *coli*’s native *leuABCD* operon. Since it was unclear whether *kivd* could be sufficiently expressed following *leuA* using this native RBS (Fig. 2, pGC3), we also cloned an alternative construct (Fig. 2, pGC4) with additional insertion of a strong RBS in front of kivd and compared their effect on alcohol production.

**Table 1:** Strains and plasmids used in this study.

Strain	Genotype	Reference
BW25113	*rrnB*_T14_ ∆*lacZ*WJ16 *hsd*R514 ∆*araBAD*_AH33_ ∆*rhaBAD*_LD78_	ref. 31
XL-1 Blue	*recA1 endA1 gyrA96 thi-1 hsdR17 supE44 relA1 lac* [F’ *proAB lacI* ^*q*^Z∆*M15 Tn10* (Tet^R^)]	Stratagene
CRS 59	BW25113/F’ [*traD36*, *proAB* ^+^, *lacI* ^q^Z∆*M15* (Tet^r^)] Δ*ilvB* Δ*ilvI* Δ*leuA*	ref. 13
**Plasmid**	**Genotype** ^**a**^	**Reference**
pCS180	P_T5_:: His6X *dddA*; pUC ori; Kan^R^	Unpublished
pSA138	P_L_lacO_1_:: *kivd yqhD*; ColE1 ori; Amp^R^	ref. 18
pSA142	P_L_lacO_1_:: *cimA*3.7 *leuABCD*; p15A ori; Kan^R^	ref. 10
pAFC52	P_L_lacO_1_:: *cimA*Δ2 *leuBCD*; p15A ori; Kan^R^	ref. 13
pGC3	P_L_lacO_1_:: *leuA* (G462D) *kivd yqhD*; ColE1 ori; Amp^R^	This study
pGC4	P_L_lacO_1_:: *leuA* (G462D) - RBS_*synthetic*_^b^ - *kivd yqhD*; ColE1 ori; Amp^R^	This study
pGC13	P_L_lacO_1_:: *leuA* (G462D) *kivd* (V461A) *yqhD*; ColE1 ori; Amp^R^	This study
pGC16	P_L_lacO_1_:: *leuA* (G462D) *kivd* (V461F) *yqhD*; ColE1 ori; Amp^R^	This study
pGC17	P_L_lacO_1_:: *leuA* (G462D) *kivd* (V461C) *yqhD*; ColE1 ori; Amp^R^	This study
pGC18	P_L_lacO_1_:: *leuA* (G462D) *kivd* (V461D) *yqhD*; ColE1 ori; Amp^R^	This study
pGC19	P_L_lacO_1_:: *leuA* (G462D) *kivd* (V461N) *yqhD*; ColE1 ori; Amp^R^	This study
pGC20	P_L_lacO_1_:: *leuA* (G462D) *kivd* (V461E) *yqhD*; ColE1 ori; Amp^R^	This study
pGC21	P_L_lacO_1_:: *leuA* (G462D) *kivd* (V461Q) *yqhD*; ColE1 ori; Amp^R^	This study
pGC22	P_L_lacO_1_:: *leuA* (G462D) *kivd* (V461G) *yqhD*; ColE1 ori; Amp^R^	This study
pGC23	P_L_lacO_1_:: *leuA* (G462D) *kivd* (V461H) *yqhD*; ColE1 ori; Amp^R^	This study
pGC24	P_L_lacO_1_:: *leuA* (G462D) *kivd* (V461L) *yqhD*; ColE1 ori; Amp^R^	This study
pGC25	P_L_lacO_1_:: *leuA* (G462D) *kivd* (V461I) *yqhD*; ColE1 ori; Amp^R^	This study
pGC26	P_L_lacO_1_:: *leuA* (G462D) *kivd* (V461K) *yqhD*; ColE1 ori; Amp^R^	This study
pGC27	P_L_lacO_1_:: *leuA* (G462D) *kivd* (V461M) *yqhD*; ColE1 ori; Amp^R^	This study
pGC28	P_L_lacO_1_:: *leuA* (G462D) *kivd* (V461P) *yqhD*; ColE1 ori; Amp^R^	This study
pGC29	P_L_lacO_1_:: *leuA* (G462D) *kivd* (V461R) *yqhD*; ColE1 ori; Amp^R^	This study
pGC30	P_L_lacO_1_:: *leuA* (G462D) *kivd* (V461S) *yqhD*; ColE1 ori; Amp^R^	This study
pGC31	P_L_lacO_1_:: *leuA* (G462D) *kivd* (V461T) *yqhD*; ColE1 ori; Amp^R^	This study
pGC32	P_L_lacO_1_:: *leuA* (G462D) *kivd* (V461W) *yqhD*; ColE1 ori; Amp^R^	This study
pGC33	P_L_lacO_1_:: *leuA* (G462D) *kivd* (V461Y) *yqhD*; ColE1 ori; Amp^R^	This study
pCSGC1	P_T5_:: His6X *kivd*; pUC ori; Kan^R^	This study
pCSGC2	P_T5_:: His6X *kivd* (V461G); pUC ori; Kan^R^	This study
pCSGC3	P_T5_:: His6X *kivd* (V461S); pUC ori; Kan^R^	This study

^a^Source of the genes is as follows: *yqhD* and *leuABCD* are from *Escherichia coli*, *kivd* is from *Lactococcus lactis*, and *cimA* is from *Methanococcus jannaschii*.

^b^Plasmid pGC4 has a synthetic RBS (AGGAGATATACC) inserted in front of *kivd* whereas pGC3 and pGC13–33 does not (*kivd* expression relies on *E*. *coli*’s native *leuB* RBS embedded at the end of *leuA*.

As shown on Fig. 2, increasing expression of LeuA^FBR^ on plasmid pGC3 effectively enhanced the relative ratio of 1-pentanol from 1% to about 40% of the total alcohol content, with an overall increase in the 1-butanol and 1-pentanol titer to 1.1 g/L and 1.0 g/L respectively in 48 h. 1-Propanol production, on the other hand, reduced by 85% to 0.4 g/L, indicating the enhanced channeling of 2KB into the ketoacid elongation cycle. Minimal level of 1-hexanol detected in the culture broth confirmed the cessation of ketoacid elongation at 2KC by WT LeuA. Overall, modification of the operon structure and copy number of key enzymes not only improved the 1-pentanol specificity, but also raised the total alcohol titer downstream of 2KB by almost three-fold, demonstrating the increased efficiency of 2-ketoacid elongation. On the other hand, existence of two RBS in tandem appeared to cause detrimental effect on Kivd expression from plasmid pGC4 as reflected by the overall drop in alcohol production. These observations suggested the sufficient expression of *kivd* by the native *leuB* RBS in *E*. *coli* and the adversity of tandem RBS for translation when placed within proximity of 10 base pairs.

### Screening of Kivd V461 variants for increased specificity toward 2-ketocaproate

Previously, several key residues delineating the Kivd active site were identified to play essential role in controlling the pocket size and acceptance of bulkier substrates, in particular F381, G402, V461, M538, and F542^4, 6, 19, 20^. Of all the Kivd variants examined for substrate specificity, V461 has been a common target selected by different studies based on the homology models and molecular simulation^4, 19, 20^. Site-directed mutagenesis of V461 to a smaller side chain alanine or a bulkier surrogate isoleucine resulted in respective shift in the substrate preference of Kivd according to pocket restriction by the different side chain. It was observed that replacing V461 with isoleucine significantly lowered the Kivd catalytic efficiency toward phenylpyruvate^19^ while substitution of V461 with alanine increased the Kivd specificity toward longer 2-ketoacids^4^. To fully explore the potential of residue 461 in controlling the substrate specificity of Kivd, we performed saturated mutagenesis of V461 and screen for increased 1-pentanol production efficiency and/or specificity. Each Kivd mutant was constructed using splicing by overlap extension (SOE) with particular codons selected based on similar frequency (~20%) as the original valine codon in the WT. Strain CRS59 (Δ*ilvB* Δ*ilvI* Δ*leuA*) transformed with pAFC52 and the plasmid harboring the individual Kivd variant was used in the screening process.

**Figure 3:**
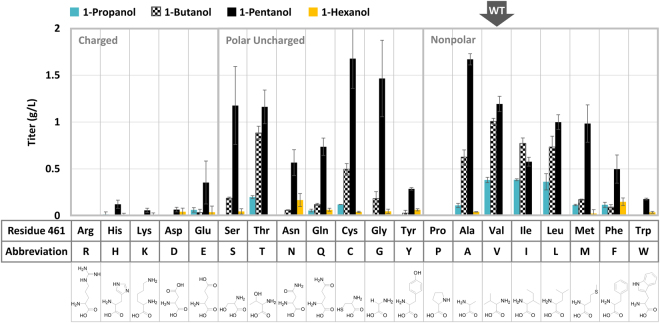
Screening of Kivd V461 variants for increased 1-pentanol specificity. Saturated mutagenesis of Kivd V461 was performed and the resulting alcohol production profile from each mutant is shown. Strain CRS59 (Δ*ilvB* Δ*ilvI* Δ*leuA*) was transformed with plasmid pAFC52 and the individual Kivd mutant harbored on plasmid pGC13–33 (or WT Kivd on pGC3). Identity of the particular amino acid substituted at V461 is shown underneath the figure along with its corresponding abbreviation and structure. The production result is grouped by the polarity of amino acid residue substituted at V461 as highlighted by the gray boxes and specification on the top left-hand corner. The alcohol production from WT Kivd is emphasized by the gray block arrow on the top. Samples were taken after 48 h of induction. The error bars represent the standard deviation of three replicates.

**Figure 4:**
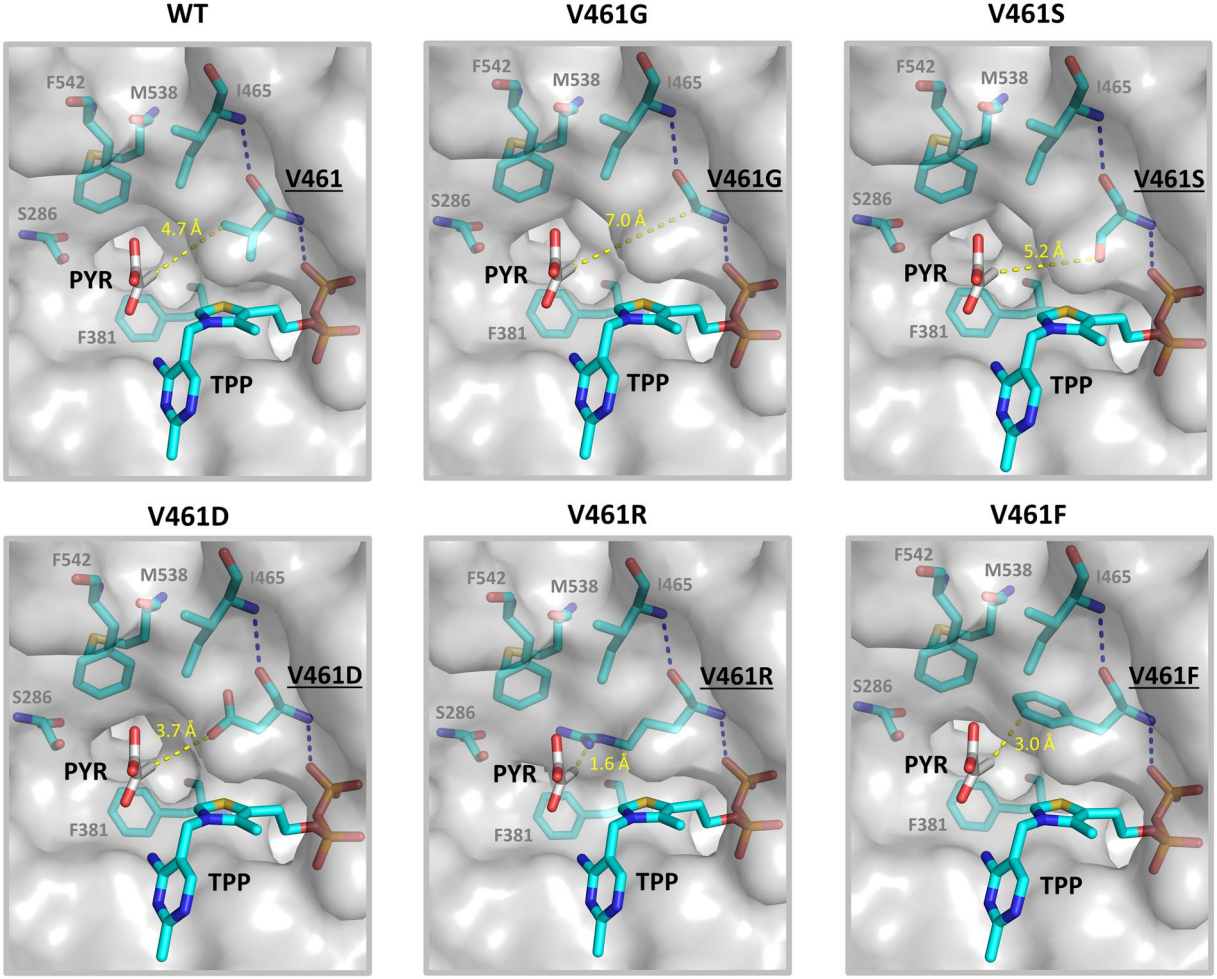
Active site of Kivd upon V461 substitution with different amino acid. The *Zymomonas mobilis* pyruvate decarboxylase (Zm-PDC, PDB: 2WVA) crystalized with pyruvate bound was aligned with the *Lactococcus lactis* Kivd (PDB: 2VBF) structure. The pyruvate molecule serves as an indicator of the putative binding position and orientation of the 2-ketoacid substrates within Kivd. Upon protein sequence alignment using Pymol and confirmation of the precise overlay of TPP and the key residues between Kivd and Zm-PDC (Supplementary Figure S1), the Zm-PDC structure was hidden, leaving only the pyruvate and the Kivd binding pocket shown. The individual mutation at residue 461 was introduced to Kivd manually in Pymol and the resulting change in pocket size is shown. The particular mutations which led to significant impact on 1-pentanol specificity and overall alcohol production are selected as the representatives here for illustration purpose. The distance between the methyl group on pyruvate and the side chain on V461 variant is shown by the dashed line and numbers in yellow. The hydrogen bonding between the V461 variant and the neighboring residue and TPP are shown by blue dashed lines. The figures were generated using Pymol v1.8.6.0. PYR, pyruvate; TPP, Thiamine pyrophosphate.

As shown on Fig. 3, a unique pattern of alcohol distribution can be derived by grouping the resulting production profile based on the polarity of each amino acid residue substituted at V461. Significant drop of straight chain alcohols to nearly non-detectable level was observed from Kivd variants containing charged side chains at the 461 position, which seemed to be independent of the side chain length. As shown by the homology model on Fig. 4, presence of charged side chains at residue 461 might have caused undesirable repulsion with the hydrophobic alkyl group on the 2-ketoacids and led to troubled entrance of the substrate. On the other hand, V461 substitution with polar but uncharged residues appeared to have much less dramatic impact on the Kivd decarboxylation activity of C4–C6 2-ketoacids as shown by the decent alcohol production. In addition to the charged residues, replacing V461 with bulky side chains consisting of aromatic rings or cyclic structure also displayed severely adverse effect on the overall alcohol production, with the lowest and highest titer observed from V461P and V461F respectively. Presence of bulky residues at position 461 might have led to reduced pocket size and restricted entrance which hindered substrate binding. As suggested by previous modeling of the Kivd active site^20^, hydrophobic interactions between the alkyl group on the 2-ketoacids and the key residues helped secure the substrate in the binding pocket. Furthermore, the substrate channel and pocket size of Kivd is restricted by the residues F542, M538, I465 and V461. These observations may help explain the significant drop in Kivd activity when V461 was replaced with charged and/or bulky residues, which possibly impaired the hydrophobic interaction and further shrunk the binding pocket respectively. As shown by Fig. 4, the modeled distance between the methyl group on pyruvate and the side chain on amino acid 461 decreased dramatically in the presence of bulky residues, indicating the potential steric clash with 2-ketoacid substrates. It is noted that the original hydrogen bonding between residue 461/465 and residue 461/cofactor TPP remains unchanged by the V461 substitutions in the homology model.

Nearly all of the successful V461 substitutions which maintained similar alcohol production efficiency and improved 1-pentanol specificity have relatively small aliphatic side chains compared to the structure of valine present in the WT, with three winners belonging in the polar group (V461S, V461C, V461G) and two winners found in the nonpolar group (V461A, V461M). Except for the case of methionine which consists of four atoms in the side chain, serine, cysteine, glycine and alanine are all short amino acids with one to two atoms in the side chain. The extra space created in the binding pocket of Kivd by these smaller amino acid substitutions might have caused loose or unstable docking of the shorter substrates 2KB and 2KV thus allowing higher specificity toward 2KC for 1-pentanol synthesis. In particular, replacing V461 with glycine resulted in the greatest distance between the methyl group on pyruvate and the side chain on residue 461, which led to the most significant improvement in the Kivd selectivity toward 2KC as shown later in the kinetic assay. It is noted that substitutions which increased 1-pentanol specificity but significantly lowered its titer, such as V461N, V461Q, V461E, and V461F, were not carried forward in this study. At the end, the best-performing Kivd variants V461S and V461G were selected from the five winners for their minimal level of 1-propanol and 1-butanol produced and were moved onto further kinetic characterization.

### Substitution V461G and V461S dramatically lowered Kivd catalytic efficiency toward 2-ketobutyrate and 2-ketovalerate

To analyze how the Kivd V461S and V461G mutants enabled significant increase in 1-pentanol production specificity, we set out to characterize their *k*_*cat*_ and *K*_*m*_ toward each 2-ketoacid (2KB, 2KV, and 2KC) in the elongation cycle using purified protein and compared the resulting level to that of the WT Kivd. Kinetic characterization of Kivd WT and mutants toward the natural substrate 2-ketoisovalerate (2KIV) was also performed to examine the effect of V461S and V461G on its catalytic efficiency for branched chain substrates. The Kivd variant V461S, V461G, and WT Kivd were re-cloned with 6X-His tag attached to the N-terminus and purified. As shown on Table 2, replacing V461 with the small and polar residue glycine and serine dramatically lowered the catalytic efficiency (*k*_*cat*_/*K*_*m*_) of Kivd toward 2KB and 2KV by 80–95% while retaining 60–90% of its original activity toward 2KC. In the case of V461G, the enhancement of 2KC specificity was majorly attributed to the decrease in *k*_*cat*_ for 2KB and increase in *K*_*m*_ for 2KV; on the other hand, the reversed phenomenon was observed in the case of V461S (decrease in *k*_*cat*_ for 2KV and increase in *K*_*m*_ for 2KB). Whereas the V461G variant demonstrated higher activity drop for 2KV, the V461S variant displayed greater activity drop for 2KB. In contrast to previous Kivd mutants V461A/F381L^4^ and G402V/M538L/F542V^6^ where increased specificity was coupled to significant decrease in the catalytic efficiency toward the target C7 2-keto-4-methylhexanoate and the C8 2-ketooctanoate respectively, in this case the V461S and V461G substitution only led to a 10–40% drop in the Kivd activity toward 2KC with slightly lowered *k*_*cat*_ and decreased *K*_*m*_. It is interesting to note that both mutants displayed about 90% drop in the catalytic efficiency toward the natural substrate 2KIV, suggesting their potential application in engineering specificity for products deriving from the branched-chain 2-ketoacids. Here, the significant reduction of Kivd activity for 2KB and 2KV by the V461G and V461S substitution allowed LeuA to pull the 2-ketoacid flux into the elongation cycle more effectively, thus increased the 1-pentanol specificity to about 90% of the total alcohol content despite the small concurrent drop of Kivd activity toward 2KC. This work also demonstrates that mutation of a single residue was able to directly tune the specificity of Kivd which previously required a combination of double or triple mutants.

**Table 2:** Kinetic study of WT Kivd and the positive Kivd mutants toward different 2-ketoacid.

*k*_*cat*_/*K*_*m*_ (M^−1^ s^−1^)	2-ketobutyrate (C4)	2-ketovalerate (C5)	2-ketocaproate (C6)	2-ketoisovalerate (isoC5)
Kivd WT	540 ± 110	1600 ± 560	840 ± 440	3100 ± 670
Kivd V461G	76 ± 36	84 ± 17	470 ± 60	220 ± 10
Kivd V461S	24 ± 15	340 ± 97	760 ± 150	380 ± 110
***k***_***cat***_ **(s** ^**−1**^ **)**	**2-ketobutyrate (C4)**	**2-ketovalerate (C5)**	**2-ketocaproate (C6)**	**2-ketoisovalerate (isoC5)**
Kivd WT	2.06 ± 0.11	4.02 ± 0.35	4.27 ± 0.64	27.8 ± 1.7
Kivd V461G	0.21 ± 0.02	0.94 ± 0.06	1.27 ± 0.04	1.30 ± 0.01
Kivd V461S	0.42 ± 0.09	0.92 ± 0.06	0.97 ± 0.04	2.99 ± 0.25
***K***_***m***_ **(mM)**	**2-ketobutyrate (C4)**	**2-ketovalerate (C5)**	**2-ketocaproate (C6)**	**2-ketoisovalerate (isoC5)**
Kivd WT	3.84 ± 0.77	2.56 ± 0.88	5.07 ± 2.55	9.04 ± 1.88
Kivd V461G	2.75 ± 1.27	11.2 ± 2.2	2.69 ± 0.33	5.92 ± 0.26
Kivd V461S	18 ± 11	2.72 ± 0.76	1.28 ± 0.25	7.86 ± 2.11

The *k*_*cat*_ and *K*_*m*_ of each Kivd variant toward the C4–C6 straight chain 2-ketoacids and the natural branched substrate 2-ketoisovalerate was measured and compared.

### Feeding of acetate enhanced supply of the iterative addition unit and increased 1-pentanol production

As shown by Fig. 1A, biosynthesis of 1-pentanol via the 2-ketoacid pathway demands high level of acetyl-CoA as the repetitive addition unit. One molecule of acetyl-CoA is consumed to extend the 2-ketoacid by one carbon in every round of the elongation cycle. Production of one molecule of 1-pentanol therefore requires three molecules of acetyl-CoA to elongate the C3 pyruvate into the C6 2KC, leading to the maximum theoretical yield of 0.5 mole 1-pentanol per mole of glucose (0.24 g/g glucose). To examine if 1-pentanol synthesis is limited by the acetyl-CoA flux, we attempted feeding of various level of acetate at different time interval to the production culture and measured the resulting alcohol accumulation. Strain Δ*ilvB* Δ*ilvI* Δ*leuA* transformed with pAFC52 and another plasmid harboring the individual Kivd variant V461S, V461G, V461A or WT Kivd was used in this study. Feeding of acetate to the production culture of WT Kivd and the well-characterized V461A mutant was performed to see if increased supply of acetyl-CoA alone could skew the alcohol distribution toward 1-pentanol.

**Figure 5:**
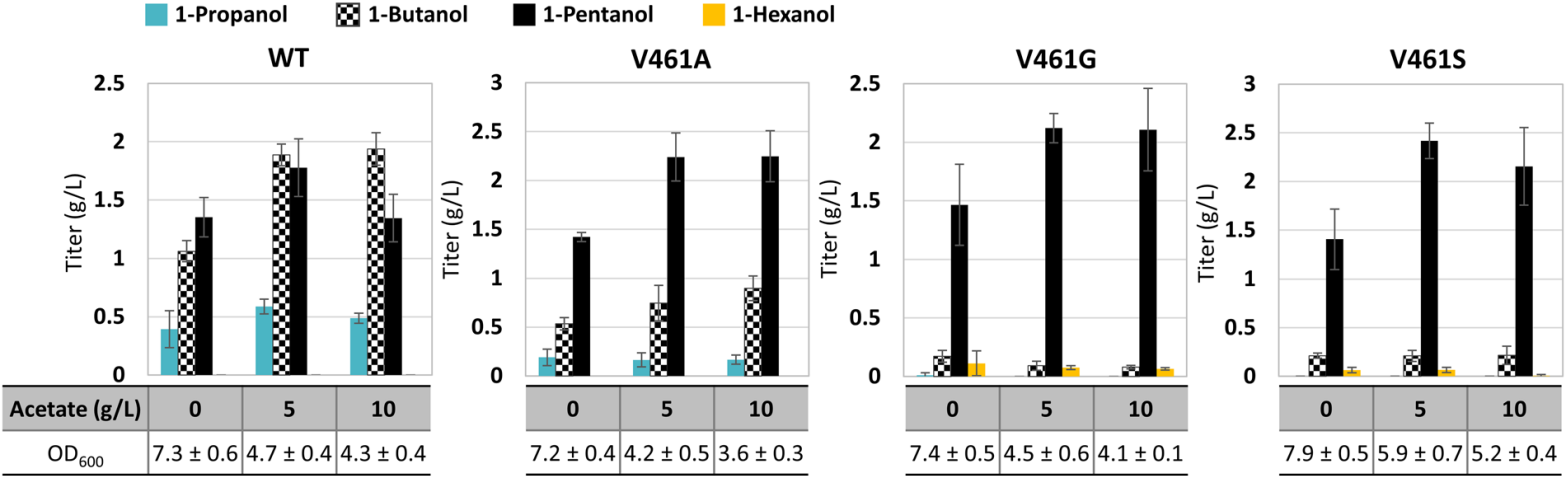
Increasing acetyl-CoA supply by acetate feeding helped driving 2-ketoacid flux into the elongation cycle. Strain CRS59 (Δ*ilvB* Δ*ilvI* Δ*leuA*) transformed with plasmid pAFC52 and pGC3 (WT Kivd), pGC22 (V461G), pGC30 (V461S), or pGC13 (V461A) were used in this study. Different level of acetate (5 or 10 g/L) was fed to the production culture at induction and the resulting alcohol distribution is compared to the one obtained without acetate feeding. Samples were taken after 48 h of induction. The error bars represent the standard deviation of three replicates.

As shown on Fig. 5, extracellular feeding of 5 g/L acetate at induction led to the most consistent improvement in 1-pentanol production from the Kivd mutant V461S and V461G, increasing the 1-pentanol titer by nearly two-fold to 2.2 g/L in 48 h. It is noted that feeding of acetate could potentially push the 1-pentanol yield above the maximum theoretical level; however, in our case the highest yield of 1-pentanol only reached about 0.12 g/g glucose. Production of 1-butanol and 1-propanol remained below 5% of the total alcohol content, indicating the efficient pulling of 2-ketoacid into the elongation cycle under enhanced supply of acetyl-CoA. On the other hand, when WT Kivd was used, elevating acetyl-CoA supply led to greater improvement in 1-butanol production (80% increase) compared to 1-pentanol (0–30% increase), thus dropping the overall 1-pentanol specificity even further. In the case of the V461A mutant, additional feeding of acetate increased the production of 1-pentanol quite significantly but was unable to boost the specificity much further due to the concurrent increase in 1-butanol synthesis. Combined with the observations above, these results demonstrate that while enhancing acetyl-CoA flux helps driving 2-ketoacids into the elongation cycle, Kivd specificity still plays a much more dominant role in determining the resulting alcohol spectrum.

**Figure 6:**
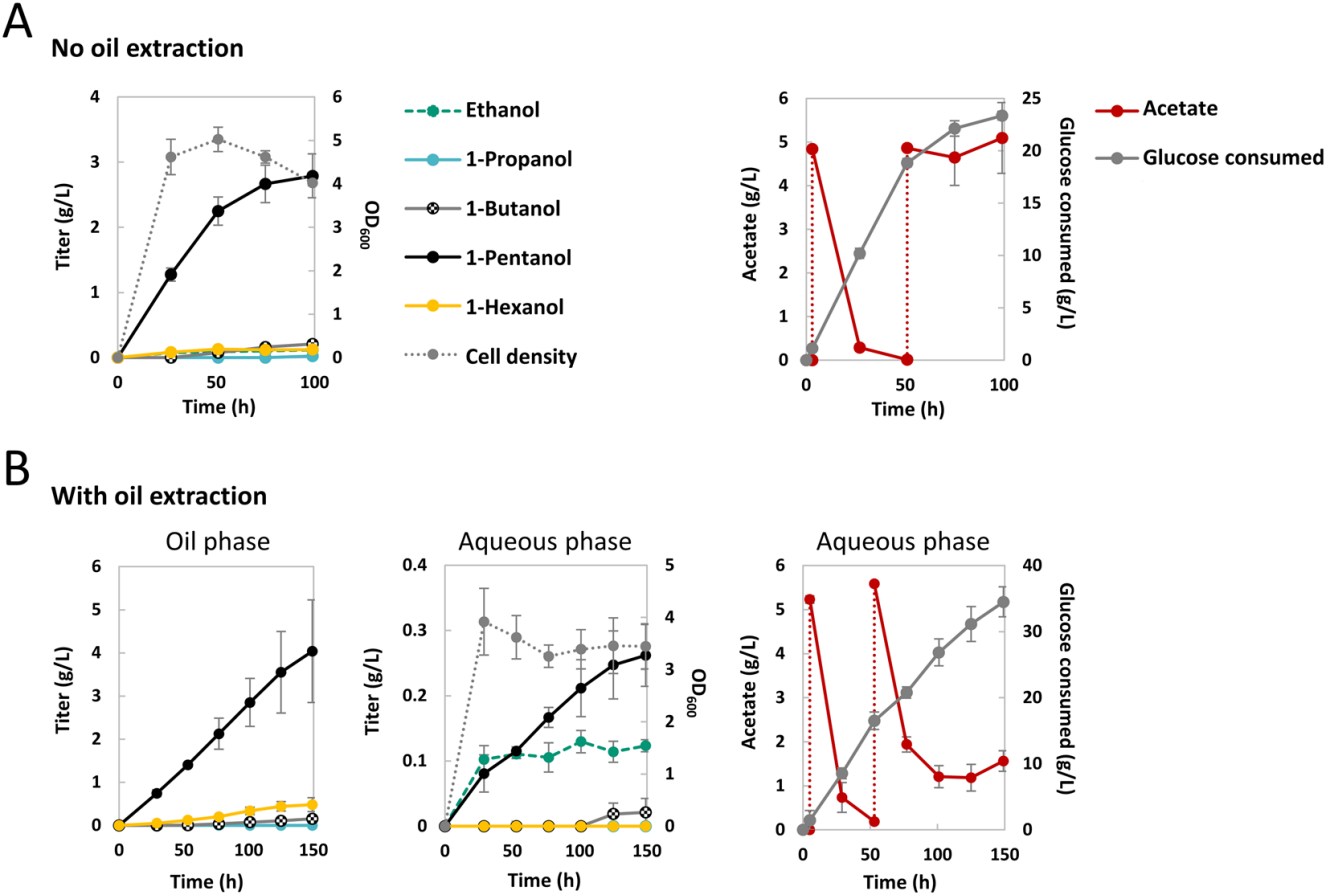
Long term production of 1-pentanol with and without *in situ* extraction using oleyl alcohol. Strain CRS59 (Δ*ilvB* Δ*ilvI* Δ*leuA*) transformed with plasmid pAFC52 and pGC22 (Kivd V461G) was used in this study. Culture pH was adjusted every day to 7 and glucose level was maintained above 10 g/L. Feeding of 5 g/L of acetate was performed at induction and at 48 h (red dashed line). (**A**) Production without oil extraction. (**B**) Production with oil extraction. Equal volume (10 mL) of oleyl alcohol was added to the culture medium at inoculation. Samples were taken every day from both the aqueous and the oil layer. Time indicates time since inoculation. The error bars represent the standard deviation of four replicates.

Our attempt to prolong and further elevate the 1-pentanol production by pH adjustment and increasing culture density was not successful. Synthesis of 1-pentanol plateaued around 2.5 g/L (Fig. 6A) despite maintenance of neutral pH, intermittent supply of acetate, and increasing concentration of yeast extract (Supplementary Figure S2A). Here, the level of yeast extract supplemented to the medium was raised above 5 g/L in the hope to sustain 1-pentanol synthesis by enhancing cellular growth and repair via increased amino acid supply. Raising the yeast extract concentration from 5 g/L to 20 g/L led to higher biomass accumulation and boosted the 1-pentanol productivity by two-fold (Supplementary Figure S2A); however, 1-pentanol yield decreased from 0.12 g/g to 0.07 g/g due to significant diversion of carbon flux into biomass. Although increasing nutrient supply doubled the production rate, it still failed to push the 1-pentanol titer beyond 2.5 g/L. These observations led us to hypothesize that 1-pentanol toxicity, which was shown to be more severe than 1-butanol and cause growth hindrance at 1 g/L^6^, contributes significantly to the cessation of 1-pentanol production here.

### *In situ* extraction using oleyl alcohol pushed 1-pentanol titer further

With good biocompatibility and low bioavailability as a microbial carbon source^21^, oleyl alcohol has been commonly used in two-phase systems to continuously remove product from the fermentation culture^22^. The effectiveness of oleyl alcohol for *in situ* extraction to minimize end-product accumulation and lower the toxicity effect has been successfully demonstrated for the production of many C4 and C5 alcohols such as 1-butanol^23–25^, isobutanol^26^, and 3-methyl-1-butanol^27^. To examine if continuous extraction of 1-pentanol would help improve the titer above 2.5 g/L, we performed two-phase production tests with equal volume of liquid culture and oleyl alcohol (10 mL each). Kivd mutant V461G was used here for its better performance in the 1-pentanol production specificity with overall lower catalytic efficiency toward 2KB and 2KV. Oleyl alcohol was added to the culture medium at inoculation to allow immediate extraction of the alcohols produced.

As shown on Fig. 6B, presence of oleyl alcohol was only able to extend the 1-pentanol production period slightly, reaching a titer of 4.3 g/L in 149 h with higher variation toward the later production phase. Affinity of alcohols for the oil layer increased with their hydrophobicity; nearly 95% and 100% of the 1-pentanol and 1-hexanol produced respectively partitioned into the oil phase. Interestingly, continuous oil extraction led to a slight decrease in the secretion of the upstream byproduct 1-butanol and slight increase of the downstream byproduct 1-hexanol, thus maintaining the 1-pentanol specificity at around 90%. Also, the addition of oleyl alcohol appeared to have a bit of detrimental effect on the alcohol productivity and cell viability, causing the 1-pentanol production rate to drop by 40% upon exposure to the oil phase compared to the level obtained without oil extraction. Similar phenomenon was also observed in the two-phase fermentation of 3-methyl-1-butanol^27^ and might be caused by the decrease in culture aerobicity upon mixing with the oil layer in screwed cap flasks. Significantly higher productivity of 1-pentanol was achieved using TB medium (Supplementary Figure S2B), although it was coupled to lower yield as a result of carbon diversion into biomass. Despite the increased production rate, 1-pentanol synthesis under the highly nutritious TB plateaued at around 4 g/L in 48 h. In all cases, we observed a positive correlation between acetate usage and 1-pentanol production; the plateau of 1-pentanol synthesis was consistently coupled to the cessation of acetate consumption.

Here, *in situ* extraction using oleyl alcohol slightly prolonged the 1-pentanol production but was unable to resolve its intrinsic pathway inefficiency as reflected by its sluggish productivity. It is noted that straight chain alcohols such as 1-pentanol and 1-hexanol were shown to induce great alteration to membrane fluidity and cellular physiology due to their deep penetration into the phospholipid structure^28–30^. In this case, continuous removal of the alcohol products may help lower the toxicity but the potential metabolic imbalance and irreversible membrane disruption caused by 1-pentanol synthesis remained as an issue. In conclusion, saturated mutagenesis of a single Kivd residue effectively improved 1-pentanol production specificity; however, production efficiency of 1-pentanol is still challenged by its toxicity and the pathway bottleneck possibly induced by the accumulation of inhibitory metabolites.

## Materials and Methods

### Reagents and chemicals

All chemicals and reagents were purchased from Sigma-Aldrich (Saint Louis, MO) or Thermo Scientific (Pittsburgh, PA) unless specified otherwise. KOD DNA polymerase was purchased from EMD Chemicals (San Diego, CA). Taq DNA ligase, Phusion High-Fidelity DNA polymerase, and T5 exonuclease were obtained from New England Biolabs (Ipswich, MA). Oligonucleotides were purchased from IDT (San Diego, CA).

### Bacterial strains

*Escherichia coli* BW25113 (*rrnB*_T14_ ∆*lacZ*_WJ16_ *hsdR514* ∆*araBAD*_AH33_ ∆*rhaBAD*_LD78_) was designated as the wild-type (WT)^31^. XL-1 Blue (Stratagene, La Jolla, CA) was used to propagate all plasmids. Construction of the strain CRS59 (BW25113 with *lacI* ^*q*^ provided on F’ with ∆*ilvB* ∆*ilvI* ∆*leuA*) was described previously^13^.

### Plasmid construction

All plasmids were created by the Gibson isothermal DNA assembly method^32^ using purified PCR fragments. A list of primers is shown on the Supplementary Table S1 and the plasmids used is shown on Table 1. It is noted that a strong RBS (AGGAGATATACC) was used to drive every gene in the synthetic operon except for *kivd* expression from pGC13-pGC33 where the native *E*. *coli* RBS for *leuB* was used (embedded at the end of *leuA*, about 10 bp away from the stop codon).

To create pGC3, the vector backbone (ColE1 ori, Amp^r^) and the operon *kivd*-*yqhD* were amplified from plasmid pSA138^18^ using primers GC1/GC2 and GC4/GC6 respectively while the feedback resistant *leuA* ^FBR^ (G462D) was amplified from plasmid pCS125 (unpublished) with primers GC3/GC5. The resulting PCR products were gel-purified and assembled together. It is noted that in the case of pGC3, the native *leuB* RBS embedded at the end of *leuA* was used to drive *kivd* expression and no synthetic RBS was inserted in front of *kivd*. To create pGC4, the operon *kivd*-*yqhD* was amplified using primers GC4/GC8 and the *leuA* ^FBR^ (G462D) was amplified using GC3/GC7. The resulting PCR products were gel-purified and assembled with the same vector backbone amplified using GC1/GC2.

To create the 19 Kivd variants with saturated mutation of the V461 residue, we designed primers carrying the specific mutation (GC47, 48, 53–88) and amplified the kivd*-*yqhD* fragment with GC4 and GC6 coupling to primers GC47-GC88 for the distinct mutation. The list of primers can be found in the Supplementary Table S1 where underline indicates the specific mutation introduced. The resulting PCR products were gel-purified and assembled with the *leuA* (G462D) fragment and the vector backbone using identical procedure. The plasmids were then sequenced to confirm the introduction of specific mutation. To create pCSGC1–3, the specific *kivd* variant was amplified using primers SW19/20 and assembled to the vector backbone amplified using primers SW21/22 from pCS180 (unpublished).

### Production medium and procedure

For all of the alcohol production experiments, single colonies were picked from LB (Luria Broth) plates and inoculated into 2 mL of LB medium contained in test tubes with the appropriate antibiotics (tetracycline 15 μg/mL, ampicillin 100 μg/mL, kanamycin 50 μg/mL). Unless otherwise specified, the overnight culture grown at 37 °C was inoculated 1% (v/v) into 15 mL of M9 medium (12.8 g/L Na_2_HPO_4_·7H_2_O, 3 g/L KH_2_PO_4_, 0.5 g/L NaCl, 1 g/L NH_4_Cl, 1 mM MgSO_4_, 1 mg/L vitamin B1 and 0.1 mM CaCl_2_) containing 30 g/L of glucose, 5 g/L of yeast extract (BD Bacto, Franklin Lakes, NJ), 1000X Trace Metal Mix A5 (2.86 g H_3_BO3, 1.81 g MnCl_2_·4H_2_O, 0.222 g ZnSO_4_·7H_2_O, 0.39 g Na_2_MoO_4_·2H_2_O, 0.079 g CuSO_4_·5H_2_O, 0.049 g Co(NO_3_)_2_·6H_2_O per liter water) and appropriate antibiotics in 250 mL screwed-cap flasks. The culture was allowed to grow at 37 °C in a rotary shaker (250 rpm) to an OD_600_ of 0.6–0.8 then induced with 0.1 mM IPTG and moved to 30 °C for alcohol production. Samples were taken throughout the next few days and culture broths were centrifuged and filtered to retrieve the supernatant. Identical procedure as described above was used in the alcohol screening experiment of the 20 different Kivd variant. Whenever feeding of acetate was performed, specified amount of sodium acetate was externally added to the production culture at induction and at various time points during the long-term production as indicated in the text.

For long-term alcohol productions lasting more than 48 h, 30 g/L of glucose was supplied initially in the production medium. Terrific Broth (12 g tryptone, 24 g yeast extract, 2.31 g KH_2_PO_4_, 12.54 g K_2_HPO_4_, 4 mL glycerol per liter of water) was also tested as a comparison. Culture pH was adjusted to 7 using 10 M NaOH every day to maintain the pH above 6.6. Additional glucose was fed at the 48 h to maintain the glucose level above 10 g/L. For the production experiments with oleyl alcohol extraction, identical procedure was used except equal volume (10 mL) of oleyl alcohol was added to the culture medium (10 mL) at inoculation. Samples were taken from both the oil and aqueous layer in equal volume upon separation of the two phases.

### Quantification of metabolites

Samples were centrifuged or filtered to gather the supernatant for GC and HPLC analysis. The amount of alcohol produced was quantified by gas chromatograph (GC) equipped with a flame ionization detector (FID). The system is a Shimadzu GC-2010 plus with an AOC-20i auto-injector and an AOC-20s auto-sampler. The separation of alcohol compounds was performed by HP-Chiral-20B column (30 m, 0.32 mm i.d., 0.25 μm film thickness) purchased from Agilent. GC oven temperature was initially held at 60 °C for 2 min and raised with a gradient of 10 °C/min until 85 °C and held for 2 min. And then it was raised with a gradient of 45 °C/min until 230 °C and held for 1 min. Helium was used as the carrier gas. The injector was maintained at 225 °C and the detector was maintained at 235 °C. The supernatant (1 μL) of culture broth was injected in split injection mode (1:15 split ratio) using isobutanol as the internal standard. For oleyl alcohol samples, identical procedure was used except samples were diluted with HPLC grade hexane to reduce the viscosity for injection accuracy.

To measure concentration of glucose and organic acids, filtered supernatant was applied to an Agilent 1260 HPLC equipped with an auto-sampler and an Agilent Hi-Plex H column (5 mM H_2_SO_4_, 0.6 mL/min, column temperature at 50 °C). Glucose was measured with refractive index detector while organic acids were detected using a photodiode array detector at 210 nm.

### Purification and *in vitro* assay of Kivd

Protein purification was done using the His-Spin Protein Miniprep Purification kit from Zymo Research (Irvine, CA). Overnight culture of the XL-1 strain harboring the target Kivd mutant (V461G and V461S) and the wild type Kivd was individually used to inoculate 20 mL of fresh LB with appropriate antibiotics. The culture was then incubated at 37 °C until exponential phase and induced with 0.1 mM IPTG. The induced culture was incubated at 30 °C for 18–24 h to allow protein expression. The overnight culture was then harvested by centrifugation and the resulting pellet was resuspended with 1 mL of binding buffer supplied in the kit. The resuspended culture was mixed with 1 mL of 0.1 mm glass beads (Biospec) and homogenated using a mini bead beater (Biospec). The soluble fraction was collected by taking the clear broth after centrifugation at 4 °C. The supernatant was run through the His-binding column, washed and eluted with buffer according to the Zymo protocol. The purified protein was collected after His-spin column purification and then mixed with glycerol for −80 °C storage. The protein concentration was determined by Bradford reagent using BSA as standards.

The *k*_*cat*_ and *K*_*m*_ of WT Kivd and the selected variants were measured for substrates 2KB, 2KV, 2KC, and 2KIV at 30 °C via a coupled assay using excess alcohol dehydrogenase (ADH) from yeast (Sigma-Aldrich A7011). The decrease of absorption at 340 nm, corresponding to the consumption of NADH, was monitored for 30 min in a 96-well plate using the Biotek Epoch 2 microplate spectrophotometer. All ketoacid substrates were dissolved in MilliQ water. Activity was measured using 1–60 mM of 2-ketoacids with the reaction mixture containing 0.5 mM NADH, 1 mM dithiothreitol, 0.2 mM TPP, 1 mM MgSO_4_, and 100 or 500 U/mL of ADH in 0.1 M potassium phosphate buffer at pH 7.4. 500 U/mL of ADH was used for all 2-ketoacids with concentration greater than 10 mM and for all reactions in the case of 2KIV. The reaction was initiated by the addition of the purified Kivd protein. A range of Kivd concentrations (30 nM to 1200 nM) was used to achieve steady state kinetics depending on the activity of each enzyme toward different 2-ketoacids. The values of *k*_*cat*_ and *K*_*m*_ were determined by fitting the initial velocity data (averaged from 2–3 repeats) to the Michaelis-Menten equation using the software Origin. The standard deviation shown on Table 2 represents error from the non-linear fitting.

### Data availability

The datasets generated and/or analyzed during the current study are available from the corresponding author on reasonable request.

## Electronic supplementary material

Supplementary Information
