# Supplementary material for: Saturated mutagenesis of ketoisovalerate decarboxylase V461 enabled specific synthesis of 1-pentanol via the ketoacid elongation cycle
Source: Sci Rep. 2017 Sep 12;7:11284. doi: 10.1038/s41598-017-11624-z (PMC5595793; doi:10.1038/s41598-017-11624-z)
Supplement: Supplementary file 1 — Supplementary Information [file 41598_2017_11624_MOESM1_ESM.doc]

**Saturated mutagenesis of ketoisovalerate decarboxylase V461 enabled specific synthesis of 1-pentanol via the ketoacid elongation cycle**

Grey S. Chen^1, #^, Siang Wun Siao^1, #^, and Claire R. Shen^1,*^

Department of Chemical Engineering, National Tsing Hua University, Hsinchu, Taiwan^1^

101, Section 2, Kuang-Fu Road, Taiwan, 30013

Phone: +886-3-5715131 (ext. 35706)

Fax: +886-3-5715408

E-mail: crshen@mx.nthu.edu.tw

^*^Corresponding author

^#^Contributed equally to this work

**Supplementary** **Table S1: Primers used in this study**

| **Primer** | **5’ → 3’ sequence** |
| --- | --- |
| GC1 | ​GGTACCTTTCTCCTCTTTAATGAATTCG |
| GC2 | TCTAGAGGCATCAAATAAAACGAAAGG |
| GC3 | TGACCGAATTCATTAAAGAGGAGAAAGGTACCATGAGCCAGCAAGTCATTATTTTC |
| GC4 | CGACTGAGCCTTTCGTTTTATTTGATGCCTCTAGATTAGCGGGCGGCTTCGTATATA |
| GC5 | TAGGTAATCTCCTACTGTATACATTCACACGGTTTCCTTGTTGTTTTC |
| GC6 | CAACAAGGAAACCGTGTGAATGTATACAGTAGGAGATTACCTATTAGACCG |
| GC7 | CTACTGTATACATGGTATATCTCCTTCACACGGTTTCCTTGTTGTTTTC |
| GC8 | GGAAACCGTGTGAAGGAGATATACCATGTATACAGTAGGAGATTACCTATTAGACCG |
| GC47 | CTTTATTATCAATAATGATGGTTATACAGCCGAAAGAGAAATTCATGGACCAAAT |
| GC48 | TGATTTGGTCCATGAATTTCTCTTTCGGCTGTATAACCATCATTATTGATAATAAAGC |
| GC53 | GCTTTATTATCAATAATGATGGTTATACATTCGAAAGAGAAATTCATGGACCA |
| GC54 | GATTTGGTCCATGAATTTCTCTTTCGAATGTATAACCATCATTATTGATAATAAAGC |
| GC55 | ATTATCAATAATGATGGTTATACATGTGAAAGAGAAATTCATGGACCAAAT |
| GC56 | TGGTCCATGAATTTCTCTTTCACATGTATAACCATCATTATTGATAATAAAGC |
| GC57 | CTTTATTATCAATAATGATGGTTATACAGACGAAAGAGAAATTCATGGACCA |
| GC58 | TGATTTGGTCCATGAATTTCTCTTTCGTCTGTATAACCATCATTATTGATAATAAAGC |
| GC59 | TATTATCAATAATGATGGTTATACAAATGAAAGAGAAATTCATGGACCAAAT |
| GC60 | TGGTCCATGAATTTCTCTTTCATTTGTATAACCATCATTATTGATAATAAAGC |
| GC61 | TATTATCAATAATGATGGTTATACAGAGGAAAGAGAAATTCATGGACCAAAT |
| GC62 | ATTTGGTCCATGAATTTCTCTTTCCTCTGTATAACCATCATTATTGATAATAAAGCA |
| GC63 | CTTTATTATCAATAATGATGGTTATACACAAGAAAGAGAAATTCATGGACCAAATC |
| GC64 | ATTTGGTCCATGAATTTCTCTTTCTTGTGTATAACCATCATTATTGATAATAAAGCA |
| GC65 | TTTATTATCAATAATGATGGTTATACAGGTGAAAGAGAAATTCATGGACCAAAT |
| GC66 | ATTTGGTCCATGAATTTCTCTTTCACCTGTATAACCATCATTATTGATAATAAAGCA |
| GC67 | TGCTTTATTATCAATAATGATGGTTATACACACGAAAGAGAAATTCATGGACCA |
| GC68 | ATTTGGTCCATGAATTTCTCTTTCGTGTGTATAACCATCATTATTGATAATAAAGCA |
| GC69 | TATTATCAATAATGATGGTTATACACTGGAAAGAGAAATTCATGGACCAAAT |
| GC70 | ATTTGGTCCATGAATTTCTCTTTCCAGTGTATAACCATCATTATTGATAATAAAGCA |
| GC71 | TGCTTTATTATCAATAATGATGGTTATACAATCGAAAGAGAAATTCATGGACCAAAT |
| GC72 | ATTTGGTCCATGAATTTCTCTTTCGATTGTATAACCATCATTATTGATAATAAAGCA |
| GC73 | TATTATCAATAATGATGGTTATACAAAGGAAAGAGAAATTCATGGACCAAAT |
| GC74 | ATTTGGTCCATGAATTTCTCTTTCCTTTGTATAACCATCATTATTGATAATAAAGCA |
| GC75 | TATTATCAATAATGATGGTTATACAATGGAAAGAGAAATTCATGGACCAAAT |
| GC76 | TGGTCCATGAATTTCTCTTTCCATTGTATAACCATCATTATTGATAATAAAGCA |
| GC77 | ATTATCAATAATGATGGTTATACACCAGAAAGAGAAATTCATGGACCAAAT |
| GC78 | ATTTGGTCCATGAATTTCTCTTTCTGGTGTATAACCATCATTATTGATAATAAAGCA |
| GC79 | CTTTATTATCAATAATGATGGTTATACACGCGAAAGAGAAATTCATGGACCA |
| GC80 | GTCCATGAATTTCTCTTTCGCGTGTATAACCATCATTATTGATAATAAAGCA |
| GC81 | TGCTTTATTATCAATAATGATGGTTATACAAGCGAAAGAGAAATTCATGGACCA |
| GC82 | TGGTCCATGAATTTCTCTTTCGCTTGTATAACCATCATTATTGATAATAAAGCA |
| GC83 | TTATTATCAATAATGATGGTTATACAACGGAAAGAGAAATTCATGGACCAAAT |
| GC84 | ATTTGGTCCATGAATTTCTCTTTCCGTTGTATAACCATCATTATTGATAATAAAGCA |
| GC85 | ATTATCAATAATGATGGTTATACATGGGAAAGAGAAATTCATGGACCAAAT |
| GC86 | ATTTGGTCCATGAATTTCTCTTTCCCATGTATAACCATCATTATTGATAATAAAGCA |
| GC87 | TTATTATCAATAATGATGGTTATACATACGAAAGAGAAATTCATGGACCA |
| GC88 | ATTTGGTCCATGAATTTCTCTTTCGTATGTATAACCATCATTATTGATAATAAAGCA |
| SW19 | TCACCATCACCATCACGGATCCGAAAACATGTATACAGTAGGAGATTACCTATTAGACC |
| SW20 | TTTATTTGATGCCTCTAGCACGCGTACCATGGTTATGATTTATTTTGTTCAGCAAATAG |
| SW21 | CTGAACAAAATAAATCATAACCATGGTACGCGTGCTAGAG |
| SW22 | TAATCTCCTACTGTATACATGTTTTCGGATCCGTGATGGT |

**Supplementary** **Figure S1: Protein sequence alignment in Pymol and confirmation of the precise overlay of TPP and the key residues between Kivd and Zm-PDC.** The *Zymomonas mobilis* pyruvate decarboxylase (Zm-PDC, PDB: 2WVA) crystalized with pyruvate bound was aligned with the *Lactococcus lactis* Kivd (PDB: 2VBF) structure. The key residues from Kivd are annotated by black font and the aligned residues from Zm-PDC are annotated by red font. The Zm-PDC structure was fainted in the background. PYR, pyruvate; TPP, Thiamine pyrophosphate.

**Supplementary Figure S2: Long term production of 1-pentanol under high supplementation of protein hydrolysates.** Strain CRS59 (Δ*ilvB* Δ*ilvI* Δ*leuA*) transformed with plasmid pAFC52 and pGC22 (Kivd V461G) was used in this study. Culture pH was adjusted every day to 7 and glucose level was maintained above 10 g/L. Feeding of 5 g/L of acetate was performed at induction and at 48 h. (A) Production using M9 medium supplemented with 20 g/L of yeast extract and no oil extraction. (B) Production using TB medium with oil extraction. Equal volume (10 mL) of oleyl alcohol was added to the culture medium at inoculation. Samples were taken every day from both the aqueous and the oil layer. YE, yeast extract; TB, terrific broth. Time indicates time since inoculation. The error bars represent the standard deviation of three independent replicates.
